# Depressive Symptoms and Sleep Disturbance in Female Nurses with Atopic Dermatitis: The Korea Nurses’ Health Study

**DOI:** 10.3390/ijerph17082743

**Published:** 2020-04-16

**Authors:** Bohye Kim, Heeja Jung, Jiyoung Kim, Jisun Lee, Oksoo Kim

**Affiliations:** 1 College of Nursing, Ewha Womans University, Seoul 03760, Korea; 2 College of Nursing, Konyang University, Daejeon 35365, Korea; 3 Department of Nursing, Sangmyung University, Cheonan-si 31066, Korea or

**Keywords:** atopic dermatitis, depression, sleep, female, nurses

## Abstract

Although the prevalence of atopic dermatitis is high in nurses, there is a lack of research on the relationship between atopic dermatitis and depressive symptoms and sleep disturbance among female nurses. This study aimed to determine the effects of atopic dermatitis on depressive symptoms and sleep disturbance in female nurses. We analyzed the data of the Korea Nurses’ Health Study, a large-scale prospective cohort study. A total of 20,613 female hospital nurses aged 20–45 years who participated in the Module 1 of Korea Nurses’ Health Study between July 2013 and November 2014 were included. The chi-square test, *t*-test, and multivariate ordinal logistic regression analysis were conducted for statistical analysis. The prevalence of atopic dermatitis among female nurses was 11.6%. The levels of depressive symptoms and sleep disturbance were higher in nurses with atopic dermatitis than those without atopic dermatitis. Nurses with atopic dermatitis were 1.16 times more likely to have depressive symptoms and 1.35 times more likely to have sleep disturbance than those without atopic dermatitis after adjusting for confounding variables. The results of this study suggest that additional support should be considered for nurses with atopic dermatitis to improve the occupational environment for managing and preventing the exacerbation of symptoms.

## 1. Introduction

Atopic dermatitis (AD) is a complex chronic inflammatory skin disease caused by interactions between genetic, immune, and environmental factors, that shows a wide range of clinical symptoms and significantly affects quality of life by increasing the burden of self-care on patients [1]. According to the Global Burden of Disease Study in 2016, about 300 million people suffer from dermatitis worldwide [2].

The prevalence of adult AD varies widely from country to country, as it is 2.7–3.1% in Korea [3,4] and 7.3% in the United States [5]. AD has a significantly higher prevalence among females (9.3–10%) than males [5,6], and the Nurses’ Health Study 2 (NHS2), which analyzed nurse health in the United States, reported that the prevalence of AD among female nurses was 10.1%, higher than that among all adults [7].

AD is known to be related to demographic characteristics such as age [8] and education level [9], and to diseases such as allergic rhinitis [10] and asthma [11]. AD is one of the leading psychophysiological disorders closely related to psychological factors like stress, anxiety, and depression and physiological factors like sleep problems [12,13]. As such, the treatment of AD requires not only care related to the enhancement and treatment of skin conditions, but also attention to, and consideration of, such psychological and physiological factors.

Many studies have reported a relationship between AD and depression [14,15] in particular, and a systematic review and meta-analysis of AD patients related to depression showed that one out of six AD patients had clinical depression, while one out of four AD patients had depressive symptoms [15]. Since the depressive symptoms experienced by AD patients are the most important risk factor for increased suicidal ideation or suicidal attempts [16], it is necessary to pay careful attention to the depressive symptoms of nurses with AD. However, depression is not uniquely related to AD patients and can be a problem for patients of other chronic inflammatory skin diseases such as psoriasis [15], so identifying the relationship between AD and depression after controlling for related variables is needed.

Many studies have also confirmed the relationship between AD and sleep disturbance by reporting that 33%, even as high as 87.1%, of adult AD patients experience sleep disturbance [17,18]. Pruritus and scratching are known to cause sleep disorders in adult AD patients [17]. AD patients are likely to experience sleep problems such as a short sleep time, trouble falling asleep, or difficulty getting up early in the morning, and such sleep problems increase fatigue. Thus, AD may lead to the risk of difficulty performing the instrumental activities of daily living, such as concentrating, remembering, eating, performing hobbies, and managing finances [18].

Nurses experience depressive symptoms at a high rate and have poor sleep quality due to shift work or long work hours [19,20]. Such depressive symptoms or sleep problems lead to an increased risk of medication errors or lower work productivity for nurses [20,21]. Despite such a high prevalence of AD, and a high rate of depressive symptoms or sleep problems in nurses, there have been few studies on the relationship between AD and depressive symptoms and sleep disturbance among nurses. Most studies of depression or sleep problems among AD patients have focused on adult patients [17,22]. The AD epidemiologic studies of female nurses conducted by the US NHS did not include depressive symptoms and investigated only cases of severe AD symptoms related to sleep disturbance [7,23].

As such, this study was intended to analyze the data of the Korea Nurses’ Health Study (KNHS), a large-scale prospective cohort study, to determine the relationships between AD prevalence and depressive symptoms and sleep disturbance level among Korean female nurses. It also examined whether AD affects depressive symptoms and sleep disturbance even when other AD-related variables are controlled for.

## 2. Materials and Methods

### 2.1. Participants

This study targeted a total of 20,613 female nurses aged 20–45 years who participated in the Module 1 questionnaire survey between July 2013 and November 2014. Of the study participants, 2393 (11.6%) were diagnosed with AD by physicians.

### 2.2. Data Collection

This study conducted by using cross-sectional survey data from the KNHS, a large-scale prospective cohort study investigating the factors that affect the health status and diseases of women of childbearing age. The KNHS is Korea’s first cohort study of women of childbearing age. KNHS started in 2013, using the questionnaires and protocols used for the Nurses’ Health Study 3 (NHS 3) in the United States, modified and supplemented according to the participants’ domestic and cultural situation [24]. Phases 1 and 2 of KNHS were conducted for six years beginning in 2013, and Phase 3 began in 2019 and will last for three years until 2021. The Phase 1 study was conducted for three years from March 2013 to December 2015, and Module 1, the initial baseline survey, was conducted from July 2013 to November 2014. The questionnaire for the KNHS Module 1 survey included questions on demographic characteristics, occupational characteristics, health behavior, illness, medication, family history, pregnancy, employment, subjective health perception, and emotional health. Although most of the questionnaire items were the same as in NHS3, some were modified and supplemented to conform to the participants’ domestic situation after discussion with the expert panel.

Depressive symptoms were measured using the Patient Health Questionnaire (PHQ-9) [25], a self-reporting tool developed to measure the level of depressive symptoms based on the diagnostic criteria of the Diagnostic and Statistical Manual of Mental Disorders, fourth edition (DSM-IV). It measures nine factors, including displeasure, fatigue, appetite change, guilt or worthlessness, decreased concentration, slow movement or restlessness, and suicidal ideation. Each question is measured on a 4-point Likert scale, with total scores ranging from 0 to 27. A higher score indicates a more depressed condition. The depressive symptoms were classified into five levels: minimal (0–4), mild (5–9), moderate (10–14), moderately severe (15–19), and severe (20–27). The cutoff point for depressive symptoms was defined as PHQ-9 ≥ 10 [25]. The sensitivity and specificity of the cutoff point of 10 points were reported to be 88% [25]. In the present study, the Cronbach’s alpha of the PHQ-9 was 0.89.

Sleep disturbance was measured using the Jenkins Sleep Questionnaire (JSQ) [26], which consists of four questions and measures the sleep problems experienced in the past four weeks: (a) trouble falling asleep; (b) waking up several times per night; (c) trouble staying asleep; and (d) waking up after the usual amount of sleep feeling tired. Each question is measured on a 6-point Likert scale from 0 (not at all) to 5 (everyday). The total scores range from 0 to 20, and a higher score indicates more experience with a sleep problem. Sleep disturbance is the case in which the mean score of each question is two points or higher, indicating participants experiencing sleep disturbance at least once a week [27,28]. As such, we regarded a total score of eight points or higher as sleep disturbance. The internal consistency coefficient was reported to be 0.79 [26]. The previous study on the validity and reliability of the Turkish version of the JSQ, used to confirm the cutoff point, reported Cronbach’s alpha to be 0.80 [28]. In the present study, the Cronbach’s alpha of the JSQ was 0.86.

The covariates of demographic characteristics, shift work, and comorbidities were adjusted based on the literature reviews to identify the effects of AD on depression and sleep disturbance. The demographic characteristics were age, education level, marital status, annual salary, and body mass index (BMI), and the comorbidities included allergic rhinitis, asthma, and psoriasis. Since shift work was confirmed to be a factor that affected depressive symptoms or sleep disturbance among nurses [19,20], it was included as one of the potential confounders. In this study, the effects of AD on depressive symptoms and sleep disturbance were analyzed using regression models. The results of the KNHS showed that depressive symptoms and sleep disturbance were related [29]. Therefore, depressive symptoms and sleep disturbance were included as confounders in both regression models.

### 2.3. Ethical Considerations

This study was reviewed and approved for research by the Institutional Review Board of the Korea Centers for Disease Control and Prevention to protect the study participants ethically (IRB No.: 2013-03CON-03-P). The study participants read the overall description of the study and guidance on the voluntariness and confidentiality of participating in the study and provided electronic informed consent before participating in the online questionnaire survey.

### 2.4. Data Analysis

The data of this study were analyzed using the SPSS 24.0 program. A chi-square test was used to examine the differences in demographic characteristics, shift work, and comorbidities between the AD group and the no AD group (Table 1). A chi-square test and *t*-test were used to examine the differences in depressive symptom level and sleep disturbance level between the AD group and the no AD group (Table 2). Lastly, a multivariate ordinal logistic regression analysis was performed to identify the effects of AD on depressive symptoms and sleep disturbance, and the results were presented using the odds ratios (ORs) and 95% confidence intervals (CIs) (Table 3 and Table 4). The threshold for statistical significance for this study was *p* < 0.05.

## 3. Results

### 3.1. Baseline Characteristics According to Atopic Dermatitis

This study targeted a total of 20,613 female nurses, 11.6% (*n* = 2393) of whom were diagnosed with AD. There were significant differences between the AD group and the no AD group according to age (χ^2^ = 60.475, *p* < 0.001), level of education (χ^2^ = 8.972, *p* = 0.011), annual salary (χ^2^ = 13.671, *p* = 0.008), marital status (χ^2^ = 43.005, *p* < 0.001), shift work (χ^2^ = 7.238, *p* = 0.007), allergic rhinitis (χ^2^ = 409.648, *p* < 0.001), asthma (χ^2^ = 63.011, *p* < 0.001), and psoriasis (χ^2^ = 505.340, *p* < 0.001) (Table 1). In the AD group, participants aged 29 years or younger made up the largest group, at 65.1%, which was higher than the 57.6% of the no AD group. The shift workers in the AD group comprised 75.8% of the sample, which was higher than the 73.2% in the no AD group, and the participants diagnosed with allergic rhinitis, asthma, and psoriasis in the AD group accounted for 34.9%, 5.1%, and 8.6%, respectively, indicating that a higher proportion of participants in the AD group had comorbidities.

### 3.2. Differences of Depressive Symptoms and Sleep Disturbance among Participants with and without AD

Table 2 shows the differences in depressive symptoms level and sleep disturbance level according to AD. The mean severity of depressive symptoms in the AD group was 8.45 ± 5.73, which was significantly higher than the 7.20 ± 5.49 in the no AD group (t = −10.117, *p* < 0.001). The mean of sleep disturbance in the AD group was 7.96 ± 5.41, which was also significantly higher than the 6.57 ± 5.17 in the no AD group (t = −11.895, *p* < 0.001). The proportion of participants with a moderate or higher level of depressive symptoms in the AD group was 35.5%, which was higher than the 26.9% in the no AD group (χ^2^ = 114.951, *p* < 0.001). The proportion of participants who experienced sleep disturbance in the AD group was also higher, at 49.2%, than the 37.7% in the no AD group (χ^2^ = 117.992, *p* < 0.001).

### 3.3. Depressive Symptoms in the Multivariate Logistic Regression

A multivariate ordinal logistic regression analysis was performed to examine the effect of AD on the depressive symptoms of participants (Table 3). Model 1 included the diagnosis of AD, and Model 2 included age, level of education, annual salary, marital status, and BMI as the confounding variables. Model 3 included shift work, and Model 4 included allergic rhinitis, asthma, psoriasis, and sleep disturbance as the confounding variables. The results of the logistic regression analysis showed that nurses with AD had 1.16 times (95% CI: 1.05–1.29, *p* < 0.01) the risk for depressive symptoms even after the demographic, shift work, and comorbidity variables were controlled. Among the confounding variables, age (under 29 years: OR = 1.56, 95% CI: 1.28–1.90; 30–39: OR = 1.27, 95% CI: 1.06–1.52), annual salary (under $20,000: OR = 1.74, 95% CI: 1.34–2.26; $20,000–$29,999: OR = 1.30, 95% CI: 1.06–1.59), marital status (single: OR = 1.57, 95% CI: 1.43–1.74), BMI (overweight: OR = 1.24, 95% CI: 1.13–1.36), shift work (OR = 1.39, 95% CI: 1.27–1.53), allergic rhinitis (OR = 1.11, 95% CI: 1.02–1.21), asthma (OR = 1.45, 95% CI: 1.19–1.78), psoriasis (OR = 1.28, 95% CI: 1.03–1.60), and sleep disturbance (OR = 6.10, 95% CI: 5.69–6.54) were significantly associated with depressive symptoms.

### 3.4. Sleep Disturbance in the Multivariate Logistic Regression

A multivariate ordinal logistic regression analysis was performed to investigate the effect of AD on sleep disturbance (Table 4). Model 1 included the diagnosis of AD, and Model 2 included age, level of education, annual salary, marital status, and BMI as the confounding variables. Model 3 included shift work, and Model 4 included allergic rhinitis, asthma, psoriasis, and depressive symptoms as the confounding variables. The results of the logistic regression analysis showed that nurses with AD had 1.35 times (95% CI: 1.23–1.49, *p* < 0.001) the risk for sleep disturbance even after the demographic, shift work, and comorbidity variables were controlled. Among the confounding variables, age (under 29 years: OR = 1.98, 95% CI: 1.68–2.35; 30–39 years: OR = 1.75, 95% CI: 1.50–2.04), annual salary ($40,000–$49,999: OR = 1.28, 95% CI: 1.08–1.51), marital status (single: OR = 1.46, 95% CI: 1.34–1.60), BMI (overweight: OR = 1.11, 95% CI: 1.02–1.21), shift work (OR = 1.63, 95% CI: 1.51–1.77), allergic rhinitis (OR = 1.20, 95% CI: 1.10–1.30), and depressive symptoms (OR = 6.10, 95% CI: 5.69–6.54) were significantly associated with sleep disturbance.

## 4. Discussion

The aim of this study was to determine AD prevalence among female nurses in Korea, and to examine whether AD affected depressive symptoms and sleep disturbance even after other confounding variables were controlled. This study showed that the prevalence of AD among Korean female nurses was 11.6%, significantly higher than the prevalence of AD among Korean adults at 2.3% [9]. This finding supports the result of the previous NHS2 study, which reported that the prevalence of AD among female nurses in the United States was 10.1%, which is higher than that among all adults [7]. It is meaningful to examine the prevalence of AD among nurses, since the prevalence of AD differs according to occupation [30] and having AD is known to affect presenteeism, overall work impairment, and activity impairment [31]. A previous study that analyzed NHS2 data reported that 57% of nurses with AD were diagnosed with it as an adult [23], suggesting that the cause of such a high prevalence of AD among nurses is the nursing job. However, this study did not include the exact time of diagnosis of AD, making it difficult to identify whether the high prevalence of AD among nurses was related to the work shift of the nurse. Therefore, future studies may consider the time of diagnosis when investigating the cause of the high prevalence of AD among nurses.

In this study, the AD group and the no AD group showed a significant difference according to age, marital status, annual salary, and shift work. The proportions of young participants aged 29 or less, and single females were particularly high among AD patients. This finding can be attributed to participants in their 20s being more likely to be single. Such results are consistent with previous studies showing that the prevalence of AD decreased as age increased [3,9,32]. The prevalence of AD in Korean adults is high in people in their 20s and tends to decrease with age, as the high prevalence of childhood and adolescence tends to be maintained in young adults [32].

This study showed that the rate of shift work was higher among the AD patients than that among the no AD patients, and it contradicted the results of the previous study, which showed that work schedule pattern was not related to AD [30]. The difference can be explained by the fact that the previous study [30] categorized the work schedule into unemployed, other time, and daytime fixed schedule.

This study also examined comorbidities. The rates of nurses with AD also having allergic rhinitis, asthma, and psoriasis were all higher than the nurses without AD. The rate of allergic rhinitis was particularly high in this study, at 34.9% of female nurses, while 49.2% of those in the United States were affected [7]. Defects in the epidermal barrier present on AD patients make it easier for environmental allergens to penetrate through the skin, causing lgE sensitization that can lead to allergic disorders such as asthma and allergic rhinitis [1]. Since nurses have a high risk of being exposed to various allergens in the hospital environment, it is necessary to examine whether allergens are related to comorbidities by investigating allergens associated with occupational characteristics of nurses with AD in the future.

The average score of depressive symptoms of participants was 8.45 points for the AD group and 7.20 points for the no AD group, indicating a significantly higher score for the AD group. The rate of participants with depressive symptoms above a moderate level was 35.5% in this study, which is higher than that of adult AD patients in the United States (11.2%) [22]. This result indicates that the depressive symptoms of female nurses with AD are at a particularly serious level. A previous study that investigated satisfaction with skin and depression levels of adult AD patients [33] reported that the AD patient group had a lower self-touching level and higher shame related to the skin than the no AD group. Because the shame associated with disease has a significant impact on quality of social relations [34], nurses with AD can easily show negative psychological results such as depression due to the occupational characteristics of continuing personal relations with patients, caregivers, and colleagues.

The result of the logistic regression analysis to measure the impact of AD on depressive symptoms showed that AD increased the risk of depressive symptoms of female nurses with AD by 1.16 times even after the confounding variables were controlled for. This finding was consistent with the previous study [22], which reported that AD of adults increased the risk of moderate depressive symptoms by 2.24 times and the risk of severe depressive symptoms by 5.64 times. Shame and lack of self-touching due to skin problems lead AD patients to have difficulties with emotion control or expression and may even lead to eventual depression or suicidal ideation [33]. Moreover, the culture of preferring smooth and flawless skin can lead to aggravation in social relationships or depression in AD patients [12]. Female AD patients are particularly sensitive to physical appearance and regard skin disease as a threat causing psychological disorder emotional disturbance, and thus they can be assumed to be more likely to experience depression than male AD patients [14]. Furthermore, in the previous qualitative research, female college students with AD reported that the itching from AD causes more distress than the pain, and they wanted to hide the wounds caused by scratching [35]. Thus, AD symptoms such as itching are considered to have an effect on depressive symptoms.

In this study, the rate of AD patients who also had sleep disturbance was 49.2%, which was significantly higher than that of the no AD group (37.7%). The study that analyzed AD cases from the NHS2 in the United States reported that the proportion of nurses who experienced sleep disturbance when AD symptoms were the worst was 40% [23]. Although a direct comparison is difficult, since this study did not investigate the degree of AD symptoms, the fact that about half of nurses in Korea and the United States experience sleep disturbance implies that the healthcare of nurses with AD should address sleep problems.

The results of the logistic regression analysis to measure the impact of AD on sleep disturbance showed that AD increased the risk of sleep disturbance of female nurses with AD by 1.35 times, even after the confounding variables were controlled for. The result is consistent with the result of the study on the relationship between AD and sleep using the 2006 National Health and Nutrition Examination Survey data in the United States from 2005 and 2006, that showed adult AD patients having 1.61 times the risk of sleep disturbance [18]. The pruritus, or scratching behaviors of AD patients, are known to be related to sleep disturbance [36]. As such, severe AD symptoms are likely to cause more sleep problems. Moreover, the risk of sleep disturbance among AD patients is likely to increase, as the low nocturnal melatonin level of AD patients is related to sleep disturbance [36], and cytokines and immune cells involved in AD development are reported to be able to interact with factors that affect sleep [37]. This study showed that allergic rhinitis was also a risk factor of sleep disturbance, as symptoms such as increased nasal congestion, itching, and mucosal edema of allergic rhinitis negatively affect sleep [38], and thus increase the risk of sleep disturbance of AD patients. Therefore, it is important to assess and intervene regarding the rhinitis symptoms for AD patients with allergic rhinitis.

Among the control variables, shift work was also an important factor that increased the risk of sleep disturbance. Since a change in cortisol level associated with the circadian rhythm can exacerbate the pruritus symptoms experienced by patients with inflammatory skin disorders [39,40], nurses with AD are more likely to experience sleep disturbance due to the worsening of symptoms related to a change in circadian rhythm because of shift work. However, this study did not investigate the type of symptoms or severity experienced as a result of AD, and it is necessary for future studies to examine the effect of shift work, including the night shift, in the relationship between AD and sleep disturbance.

In this study, depressive symptoms and sleep disturbance increased the risk of occurrence of the other. This supports the finding of a relationship between depressive symptoms and sleep disturbance in previous studies conducted with nurses and nursing students [29,40]. Major depressive symptoms and sleep quality are bidirectional [41]. Women experienced longer sleep-onset latency and low sleep quality when they had depression and, conversely, more anhedonic depression when sleep quality was low [42]. Therefore, it is important to evaluate depressive symptoms and sleep problems together and consider them as risk factors to each other. Cognitive-Behavioral Therapy, such as mindfulness-based intervention or art therapy, has been reported to improve depression [43]. Therefore, it can be applied to planning interventions to reduce the depressive symptoms of nurses with AD. Because pruritus management is required for sleep improvement in AD patients [17], it should be included in identifying itch-scratch triggers and using methods to replace scratching or use relaxation techniques [44].

There are a few limitations of this study. This study was a cross-sectional study that could not explain the causal relationship between variables. Therefore, future longitudinal studies to investigate the causal relationship are needed. In this study, we did not consider the time of AD diagnosis. It is necessary for future studies to investigate if there is any change in depressive symptoms or sleep disturbance according to whether AD was developed during childhood or adulthood, and whether it developed after the subject began working as a nurse. This study did not investigate the sites where AD symptoms developed or the severity and treatment of AD symptoms. It also did not investigate nurses’ working characteristics, such as the degree of exposure to disinfectants, which could affect the manifestation of AD symptoms. More studies should be conducted to analyze the impact of depressive symptoms and sleep disturbance with consideration of the factors that affect the characteristics and manifestation of symptoms of nurses with AD. This study did not include information about history or treatment for depression or sleep disorder before AD diagnosis. Therefore, it is necessary to consider these factors in future studies.

## 5. Conclusions

This study showed that the AD prevalence of Korean female nurses was 11.6% and confirmed that the levels of depressive symptoms and sleep disturbance were higher among female nurses with AD than those without AD. AD may increase the likelihood of the risk of depressive symptoms and sleep disturbance of female nurses even after the confounding variables, such as demographics, comorbidities, and shift work, are controlled for.

While previous studies on the relationships between AD and depressive symptoms and sleep problems mainly targeted adult AD patients, this study is significant in that it presented the importance of the depressive symptoms and sleep disturbance problems of female nurses with AD by looking at the relationship between variables. The results imply the need for more careful monitoring and intervention of depressive symptoms and sleep disturbance problems for female nurses with AD.

## Figures and Tables

**Table 1:** Baseline characteristics according to atopic dermatitis (*N* = 20,613).

Characteristics	Category	No AD (*N* = 18,220)		AD (*N* = 2393)		*x^2^*	*p*
		*n*	%	*n*	%		
Age, years	≤29	10,496	57.6	1559	65.1	60.475	<0.001
	30–39	6136	33.7	706	29.5		
	≥40	1588	8.7	128	5.3		
Level of education (*n* = 20,612)	Master or higher	1377	7.6	148	6.2	8.972	0.011
	3-year college	8661	47.5	1110	46.4		
	4-year college	8181	44.9	1135	47.4		
Annual salary (*n* = 20,611)	≥$50,000	1191	6.5	135	5.6	13.671	0.008
	$40,000–$49,999	2339	12.8	283	11.8		
	$30,000–$39,999	6859	37.6	872	36.4		
	$20,000–$29,999	7239	39.7	1002	41.9		
	<$20,000	590	3.2	101	4.2		
Marital status (*n* = 20,611)	Single	11,919	65.4	1727	72.2	43.005	<0.001
	Married	6299	34.6	666	27.8		
BMI (*n* = 20,520)	Normal (18.5–23 kg/m^2^)	11,886	65.5	1546	64.8	1.432	0.489
	Underweight (<18.5 kg/m^2^)	2875	15.9	372	15.6		
	Overweight (≥23 kg/m^2^)	3373	18.6	468	19.6		
Shift-work (*n* = 20,611)	No	4886	26.8	580	24.2	7.238	0.007
	Yes	13,332	73.2	1813	75.8		
Allergic rhinitis	No	15,044	82.6	1559	65.1	409.648	<0.001
	Yes	3176	17.4	834	34.9		
Asthma	No	17,801	97.7	2272	94.9	63.011	<0.001
	Yes	419	2.3	121	5.1		
Psoriasis	No	17,969	98.6	2188	91.4	505.340	<0.001
	Yes	251	1.4	205	8.6		

AD, atopic dermatitis; BMI, body mass index.

**Table 2:** Differences in depressive symptoms and sleep among subjects with and without atopic dermatitis (*N* = 20,613).

Variables		No AD (*N* = 18,220)		AD (*N* = 2393)		t or *x*^2^	*p*
		*n*	%	*n*	%		
Depressive symptoms (*n* = 20,586)	M ± SD	7.20 ± 5.49		8.45 ± 5.73		−10.117	<0.001
Minimal		6644	36.5	647	27.1	114.951	<0.001
Mild		6667	36.6	894	37.4		
Moderate		2899	15.9	489	20.5		
Moderately severe		1430	7.9	242	10.1		
Severe		557	3.1	117	4.9		
Sleep (*n* = 20,610)	M ± SD	6.57 ± 5.17		7.96 ± 5.41		−11.895	<0.001
No sleep disturbance		11,353	62.3	1215	50.8	117.992	<0.001
Sleep disturbance		6865	37.7	1177	49.2		

AD, atopic dermatitis; M, mean; SD, standard deviation.

**Table 3:** Odds and 95% confidence interval for depressive symptoms in the multivariate logistic regression analysis (*N* = 20,613).

Variables	Model 1		Model 2		Model 3		Model 4	
	OR	95% CI	OR	95% CI	OR	95% CI	OR	95% CI
Atopic dermatitis								
No	Ref.		Ref.		Ref.		Ref.	
Yes	1.50 ***	1.37–1.64	1.40 ***	1.28–1.54	1.41 ***	1.28–1.54	1.16 **	1.05–1.29
Age, years								
≥40			Ref.		Ref.		Ref.	
≤29			2.43 ***	2.02–2.92	2.01 ***	1.67–2.43	1.56 ***	1.28–1.90
30–39			1.74 **	1.46–2.06	1.56 **	1.31–1.85	1.27 *	1.06–1.52
Level of education								
Master or higher			Ref.		Ref.		Ref.	
3-year college			1.09	0.93–1.27	1.01	0.86–1.18	0.98	0.83–1.16
4-year college			1.09	0.94–1.28	1.03	0.88–1.21	1.02	0.86–1.20
Annual salary								
≥$50,000			Ref.		Ref.		Ref.	
<$20,000			1.36 *	1.07–1.74	1.60 ***	1.25–2.05	1.74 ***	1.34–2.26
$20,000–$29,999			1.18	0.98–1.43	1.27 *	1.05–1.53	1.30 *	1.06–1.59
$30,000–$39,999			1.23 *	1.02–1.48	1.27 *	1.06–1.52	1.22	1.00–1.48
$40,000–$49,999			1.18	0.97–1.43	1.17	0.96–1.42	1.05	0.85–1.29
Marital status								
Married			Ref.		Ref.		Ref.	
Single			1.98 ***	1.80–2.16	1.84 ***	1.68–2.02	1.57 ***	1.43–1.74
BMI								
Normal (18.5–23 kg/m^2^)			Ref.		Ref.		Ref.	
Underweight (<18.5 kg/m^2^)			1.11 *	1.02–1.20	1.10 *	1.01–1.20	1.10	1.00–1.20
Overweight (≥23 kg/m^2^)			1.31 ***	1.20–1.42	1.30 ***	1.19–1.41	1.24 ***	1.13–1.36
Shift work								
No					Ref.		Ref.	
Yes					1.70 ***	1.56–1.84	1.39 ***	1.27–1.53
Allergic rhinitis								
No							Ref.	
Yes							1.11 *	1.02–1.21
Asthma								
No							Ref.	
Yes							1.45 ***	1.19–1.78
Psoriasis								
No							Ref.	
Yes							1.28 *	1.03–1.60
Sleep disturbance								
No							Ref	
Yes							6.10 ***	5.69–6.54
Nagelkerke R^2^	0.005 ***		0.072 ***		0.082 ***		0.260 ***	
χ^2^/df	75.364/1		1042.903/12		1200.214/13		4075.853/17	

*** *p* < 0.001, ** *p* < 0.01, * *p* < 0.05. OR, odds ratio; 95% CI, 95% confidence interval; Ref, reference group; BMI, body mass index.

**Table 4:** Odds and 95% confidence interval for sleep disturbance in the multivariate logistic regression analysis (*N* = 20,613).

Variables	Model 1		Model 2		Model 3		Model 4	
	OR	95% CI	OR	95% CI	OR	95% CI	OR	95% CI
Atopic dermatitis								
No	Ref.		Ref.		Ref.		Ref.	
Yes	1.60 ***	1.47–1.74	1.51 ***	1.38–1.65	1.52 ***	1.39–1.66	1.35 ***	1.23–1.49
Age, years								
≥40			Ref.		Ref.		Ref.	
≤29			2.79 ***	2.39–3.27	2.26 ***	1.93–2.66	1.98 ***	1.68–2.35
30–39			2.09 ***	1.81–2.41	1.85 ***	1.60–2.14	1.75 ***	1.50–2.04
Level of education								
Master or higher			Ref.		Ref.		Ref.	
3-year college			1.16 *	1.01–1.33	1.06	0.93–1.22	1.07	0.93–1.24
4-year college			1.11	0.97–1.27	1.04	0.91–1.19	1.03	0.89–1.19
Annual salary								
≥$50,000			Ref.		Ref.		Ref.	
<$20,000			0.84	0.68–1.05	1.02	0.81–1.27	0.83	0.65–1.06
$20,000–$29,999			0.94	0.80–1.10	1.02	0.87–1.20	0.93	0.79–1.10
$30,000–$39,999			1.13	0.97–1.32	1.18 *	1.01–1.37	1.11	0.94–1.30
$40,000–$49,999			1.31 **	1.11–1.54	1.30 **	1.10–1.52	1.28 **	1.08–1.51
Marital status								
Married			Ref.		Ref.		Ref.	
Single			1.83 ***	1.69–1.98	1.69 ***	1.56–1.84	1.46 ***	1.34–1.60
BMI								
Normal (18.5–23 kg/m^2^)			Ref.		Ref.		Ref.	
Underweight (<18.5 kg/m^2^)			1.05	0.97–1.14	1.04	0.96–1.13	1.01	0.92–1.10
Overweight (≥23 kg/m^2^)			1.21 ***	1.12–1.31	1.20 ***	1.11–1.30	1.11 *	1.02–1.21
Shift work								
No					Ref.		Ref.	
Yes					1.81 ***	1.68–1.95	1.63 ***	1.51–1.77
Allergic rhinitis								
No							Ref.	
Yes							1.20 ***	1.10–1.30
Asthma								
No							Ref.	
Yes							1.10	0.90–1.34
Psoriasis								
No							Ref.	
Yes							1.17	0.95–1.45
Depressive symptoms								
Minimal~mild							Ref.	
Moderate~severe							6.10 ***	5.69–6.54
Nagelkerke R^2^	0.007 ***		0.072 ***		0.088 ***		0.254 ***	
χ^2^/df	113.315/1		1120.635/12		1370.157/13		4244.053/17	

*** *p* < 0.001, ** *p* < 0.01, * *p* < 0.05. OR, odds ratio; 95% CI, 95% confidence interval; Ref, reference group; BMI, body mass index.

## References

[1] Nutten S. (2015). Atopic dermatitis: Global epidemiology and risk factors. Ann. Nutr. Metab..

[2] Vos T., Abajobir A.A., Abate K.H., Abbafati C., Abbas K.M., Abd-Allah F., Abdulkader R.S., Abdulle A.M., Abebo T.A., Abera S.F. (2017). Global, regional, and national incidence, prevalence, and years lived with disability for 328 diseases and injuries for 195 countries, 1990-2016: A systematic analysis for the Global Burden of Disease Study 2016. Lancet.

[3] Ko S.J., Hong H.S. (2016). Prevalence and factors influencing atopic dermatitis in Korean adults in relation to Vitamin D levels: Data from the Korea National Health and Nutrition Examination Survey 2008-2013. Int. J. Bio-Sci. Bio-Technol..

[4] Lee J.S., Kim J.M., Seok J., Kim B.J. (2017). Correlation between socio-economic status and atopic dermatitis in Korean adults: The Korea National Health and Nutrition Examination Survey (2007–2014). J. Eur. Acad. Dermatol. Venereol..

[5] Chiesa Fuxench Z.C., Block J.K., Boguniewicz M., Boyle J., Fonacier L., Gelfand J.M., Grayson M.H., Margolis D.J., Mitchell L., Silverberg J.I. (2019). Atopic dermatitis in America Study: A cross-sectional study examining the prevalence and disease burden of atopic dermatitis in the US adult population. J. Investig. Dermatol..

[6] Barbarot S., Auziere S., Gadkari A., Girolomoni G., Puig L., Simpson E.L., Margolis D.J., De Bruin-Weller M., Eckert L. (2018). Epidemiology of atopic dermatitis in adults: Results from an international survey. Allergy.

[7] Drucker A.M., Li W.Q., Lin L., Cho E., Li T., Camargo C.A., Qureshi A.A. (2016). Atopic dermatitis (eczema) in US female nurses: Lifestyle risk factors and atopic comorbidities. Br. J. Dermatol..

[8] Sacotte R., Silverberg J.I. (2018). Epidemiology of adult atopic dermatitis. Clin. Dermatol..

[9] Kim B.J., Jung J.-A., Lee J.S. (2015). Association between social economic status and atopic dermatitis in Korean adult: An analysis of the fifth Korea National Health and Nutrition Examination Survey (2010-2012). Allergy Asthma Respir. Dis..

[10] Eichenfield L.F., Stein Gold L.F. (2017). The disease burden of atopic dermatitis. Semin. Cutan. Med. Surg..

[11] Arima K., Gupta S., Gadkari A., Hiragun T., Kono T., Katayama I., Demiya S., Eckert L. (2018). Burden of atopic dermatitis in Japanese adults: Analysis of data from the 2013 National Health and Wellness Survey. J. Dermatol..

[12] Senra M.S., Wollenberg A. (2014). Psychodermatological aspects of atopic dermatitis. Br. J. Dermatol..

[13] Jafferany M., Patel A. (2019). Understanding psychocutaneous disease: Psychosocial & psychoneuroimmunologic perspectives. Int. J. Dermatol..

[14] Mina S., Jabeen M., Singh S., Verma R. (2015). Gender differences in depression and anxiety among atopic dermatitis patients. Indian J. Dermatol..

[15] Patel K.R., Immaneni S., Singam V., Rastogi S., Silverberg J.I. (2019). Association between atopic dermatitis, depression, and suicidal ideation: A systematic review and meta-analysis. J. Am. Acad. Dermatol..

[16] Keilp J.G., Ellis S.P., Gorlyn M., Burke A.K., Oquendo M.A., Mann J.J., Grunebaum M.F. (2018). Suicidal ideation declines with improvement in the subjective symptoms of major depression. J. Affect. Disord..

[17] Jeon C., Yan D., Nakamura M., Sekhon S., Bhutani T., Berger T., Liao W. (2017). Frequency and management of sleep disturbance in adults with atopic dermatitis: A systematic review. Dermatol. Ther..

[18] Yu S.H., Attarian H., Zee P., Silverberg J.I. (2016). Burden of sleep and fatigue in US adults with atopic dermatitis. Dermatitis.

[19] Lee H.Y., Kim M.S., Kim O., Lee I.H., Kim H.K. (2016). Association between shift work and severity of depressive symptoms among female nurses: The Korea Nurses’ Health Study. J. Nurs. Manag..

[20] Park E., Lee H.Y., Park C.S.-Y. (2018). Association between sleep quality and nurse productivity among Korean clinical nurses. J. Nurs. Manag..

[21] Melnyk B.M., Orsolini L., Tan A., Arslanian-Engoren C., Melkus G.D., Dunbar-Jacob J., Rice V.H., Millan A., Dunbar S.B., Braun L.T. (2018). A national study links nurses’ physical and mental health to medical errors and perceived worksite wellness. J. Occup. Environ. Med..

[22] Yu S.H., Silverberg J.I. (2015). Association between atopic dermatitis and depression in US adults. J. Investig. Dermatol..

[23] Drucker A.M., Cho E., Li W.Q., Camargo C.A., Li T., Qureshi A.A. (2019). Diagnosis validation and clinical characterization of atopic dermatitis in Nurses’ Health Study 2. J. Eur. Acad. Dermatol. Venereol..

[24] Kim O., Ahn Y., Lee H.Y., Jang H.J., Kim S., Lee J.E., Jung H., Cho E., Lim J.Y., Kim M.J. (2017). The Korea Nurses’ Health Study: A prospective cohort study. J. Womens Health.

[25] Kroenke K., Spitzer R.L., Williams J.B.W. (2001). The PHQ-9: Validity of a brief depression severity measure. J. Gen. Intern. Med..

[26] Jenkins C.D., Stanton B.-A., Niemcryk S.J., Rose R.M. (1988). A scale for the estimation of sleep problems in clinical research. J. Clin. Epidemiol..

[27] Schubert C.R., Cruickshanks K.J., Dalton D.S., Klein B.E.K., Klein R., Nondahl D.M. (2002). Prevalence of sleep problems and quality of life in an older population. Sleep.

[28] Duruoz M.T., Unal C., Ulutatar F., Sanal Toprak C., Gunduz O.H. (2018). The validity and reliability of Turkish version of the Jenkins Sleep Evaluation Scale in rheumatoid arthritis. Arch. Rheumatol..

[29] Kim O., Kim M.S., Kim J., Lee J.E., Jung H. (2018). Binge eating disorder and depressive symptoms among females of child-bearing age: The Korea Nurses’ Health Study. BMC Psychiatry.

[30] Kwak Y., Kim Y. (2017). Associations between prevalence of adult atopic dermatitis and occupational characteristics. Int. J. Nurs. Pract..

[31] Eckert L., Gupta S., Gadkari A., Mahajan P., Gelfand J.M. (2019). Burden of illness in adults with atopic dermatitis: Analysis of National Health and Wellness Survey data from France, Germany, Italy, Spain, and the United Kingdom. J. Am. Acad. Dermatol..

[32] Im D., Yang Y.S., Choi H.R., Choi S., Nahm H., Han K., Hong S.-C., Kim J.K., Cho J.H. (2017). Prevalence of allergic disease in Korean adults: Results from the Korea National Health and Nutrition Examination Survey (2010–2012). Korean J. Otorhinolaryngol.-Head Neck Surg..

[33] Dieris-Hirche J., Gieler U., Petrak F., Milch W., Te Wildt B., Dieris B., Herpertz S. (2017). Suicidal ideation in adult patients with atopic dermatitis: A German cross-sectional study. Acta. Derm. Venereol..

[34] Trindade I.A., Duarte J., Ferreira C., Coutinho M., Pinto-Gouveia J. (2018). The impact of illness-related shame on psychological health and social relationships: Testing a mediational model in students with chronic illness. Clin. Psychol. Psychother..

[35] Oh Y.H. (2015). Life experience of female college student with atopic dermatitis. J. Korea Contents Assoc..

[36] Chang Y.S., Chou Y.T., Lee J.H., Lee P.L., Dai Y.S., Sun C., Lin Y.T., Wang L.C., Yu H.H., Yang Y.H. (2014). Atopic dermatitis, melatonin, and sleep disturbance. Pediatrics.

[37] Chang Y.S., Chiang B.L. (2016). Mechanism of sleep disturbance in children with atopic dermatitis and the role of the circadian rhythm and melatonin. Int. J. Mol. Sci..

[38] Correia C., Baroody F.M., Fishbein A., Sheldon S.H. (2019). Allergic rhinitis and sleep: Approaches to management. Allergy and Sleep: Basic Principles and Clinical Practice.

[39] Gupta M.A., Gupta A.K. (2013). Sleep-wake disorders and dermatology. Clin. Dermatol..

[40] Kim K.H., Hwang E.H. (2018). Correlation among insomnia, sleep quality, depression, and circadian rhythm in nursing baccalaureate students. J. Korean Pubilc Health Nurs..

[41] Rothe N., Schulze J., Kirschbaum C., Buske-Kirschbaum A., Penz M., Wekenborg M.K., Walther A. (2020). Sleep disturbances in major depressive and burnout syndrome: A longitudinal analysis. Psychiatry Res..

[42] Kalmbach D.A., Arnedt J.T., Swanson L.M., Rapier J.L., Ciesla J.A. (2017). Reciprocal dynamics between self-rated sleep and symptoms of depression and anxiety in young adult women: A 14-day diary study. Sleep Med..

[43] Lee J., Kim H., Lim S. (2019). An integrative review of job stress and mental health intervention programs for experienced nurses. J. Korean Acad. Psychiatr. Ment. Health Nurs..

[44] Spielman S.C., LeBovidge J.S., Timmons K.G., Schneider L.C. (2015). A Review of multidisciplinary interventions in atopic dermatitis. J. Clin. Med..

